# Acute Appendicitis
*A Bristol University Post-graduate Lecture.


**Published:** 1939

**Authors:** H. Chitty

**Affiliations:** Surgeon, Bristol Royal Infirmary


### ACUTE APPENDICITIS.*

BY

H. Chitty, M.S., F.R.C.S.,

*Surgeon, Bristol Royal Infirmary.*

Appendicitis continues to exact a heavy toll of human life. The cases that reach the post-mortem room have almost all of them been operated on late, when abscess or peritonitis had developed. Improved technique will doubtless save some of these, but it seems to me that our chief hope rests in earlier diagnosis and operation. Patients often fail to consult a medical man in the earliest stage of their illness, but all too often the fault lies with the medical man.

### Ætiology.

That appendicitis has become much more common in the last half century is indubitable, but which of the suggested causes—diet, habits, presence of infective agents, etc.—are responsible for this I have, frankly, no idea. Concerning the increased frequency of the disease, the unpublished observations of the late Dr. Statham, of Cheddar, are very convincing. For many years before his death, he had been encountering a fair number of cases in his practice and, convinced that he had not met similar cases when he first set up in practice some fifty years previously, he looked up the careful notes which he had made on every

* A Bristol University Post-graduate Lecture.

one of the patients whom he had attended in the first ten years of his professional career. In only one instance could he find symptoms in the least suggestive of a mild attack of appendicitis.

Appendicitis may occur at any age; my own earliest case was an appendix abscess in a child of eighteen months and my oldest a case of suppurative appendicitis in the hernial sac of a man in his nineties. The disease is, however, rare during the first five years of life. The vast majority of cases are met with between the ages of five and twenty-five, after which it becomes gradually less and less frequent.

### PATHOLOGY.

We are indebted to the late Professor D. P. D. Wilkie for pointing out that under the title "Appendicitis" we include two very dissimilar pathological entities. One is an inflammation of varying severity beginning in the mucous membrane and lymphoid tissue of the appendix and later involving the muscular wall and peritoneum. The tendency is for such a condition to recover spontaneously; at the worst it is unlikely to give rise to more than a localized abscess. It may, however, produce permanent changes in the appendix, for ulceration of the mucous membrane or inflammation of the muscular wall may lead to the formation of a fibrous stricture, whilst shortening of the meso-appendix or peritoneal adhesions may cause kinking of the appendix. These conditions, by interfering with the normal emptying of the appendix, may give rise to recurrent attacks of appendicular colic or to appendicular obstruction.

The second is obstruction of the appendix leading to gangrene. The failure of the appendix completely to empty itself often leads to the formation of a fæcolith, which, becoming impacted in the narrow lumen, may precipitate an attack of appendicular obstruction. When the distal part of the appendix is unable to evacuate its contents it becomes distended by its own secretion, and virulent pathogenic organisms rapidly multiply. The stretching of the appendix wall, aided by the absorption of toxins under pressure, soon produces gangrene of the appendix; rupture may take place within twenty-four hours. Meanwhile there has been no time for defensive adhesions to form, so that when rupture occurs the general peritoneal cavity is flooded by a quantity of pus and mixed pathogenic organisms. It is the obstructed appendix which is responsible for almost the whole of the mortality associated with appendicitis.

### HISTORY.

Even at the present day when medical men are much more surgically-minded than ever before, comparatively few cases of appendicitis are seen by a surgeon in their first attack. The great majority when they come into his hands give a history of previous bouts of abdominal pain of a character similar to the one for which he sees them. The earlier attacks have not as a rule been diagnosed as appendicitis, but have been labelled gastritis, bilious attacks, indigestion or gastric influenza. It is surprising how few doctors dare to diagnose appendicitis until obvious signs of peritonitis are present. Yet only by their so doing is it likely that there will be any great diminution in the death-roll.

### SIGNS AND SYMPTOMS.

Professor Wilkie used to maintain that the inflammatory and obstructive types of appendicitis could be clinically differentiated. It is frequently possible to do this, but it is certainly not invariably the case. The typical inflammatory attack commences with a persistent abdominal pain, though the patient may have felt "out of sorts" for a day or two beforehand. At first the pain is commonly referred to the epigastrium or umbilicus, but it soon settles down into the right iliac fossa. Vomiting may occur, but is rarely a distressing feature. The tongue is furred and the temperature slightly raised, though it is seldom above 100° F. in the first twenty-four hours. There is a corresponding rise in the pulse rate. Tenderness is usually present on deep pressure over the appendix. In this connection it is important to remember that the position of the appendix is very variable. If no tenderness can be elicited in the right iliac fossa a retrocæcal appendix should be sought for by pressing deeply over the ascending colon, and if this proceeding is painless, then a pelvic examination must be made, lest the appendix should be lying below the pelvic brim. If no tenderness can be elicited, yet the symptoms are otherwise suggestive of appendicitis, the same careful examination must be repeated a few hours later.

In obstructive appendicitis the symptoms resemble those of an intestinal obstruction rather than those of an inflammatory lesion. Pain recurs in spasms like those of a severe abdominal colic; vomiting is often prolonged and distressing. The tongue is furred, the temperature remains normal (until the onset of peritonitis) or may even be sub-normal. Tenderness can usually be elicited when pressure is made over the appendix, wherever it may happen to be. After some hours, the vomiting sometimes ceases and the pain becomes less severe. The patient may seem altogether better, and the medical attendant may feel justified in postponing any consideration of operation, under the impression that the attack is subsiding spontaneously. Such an improvement, however, is frequently due to paralysis of the muscles of the appendix wall owing to the onset of gangrene. It may suddenly be followed by perforation, with a recurrence of pain and tenderness, a rising pulse and temperature, and the signs of acute peritonitis — "guarding" and rigidity of the abdominal wall and dullness on percussion over the lower part of the abdomen. In the case of a pelvic appendix these signs may be absent, but extreme tenderness is elicited on rectal examination.

Among other signs and symptoms, constipation is the rule, but diarrhœa is present in 10 per cent. of the cases, particularly when the appendix is low down in the pelvis. In such cases dysuria is also commonly present. One frequently finds that a patient keeps the thighs flexed and may complain of pain when the right thigh is extended. This is especially common where the appendix lies on the ilio-psoas muscle. It is a particularly valuable sign in children. In some cases where deep pressure causes very little discomfort, the sudden removal of the hand will give rise to acute pain.

### DIFFERENTIAL DIAGNOSIS.

A right basal pneumonia may lead to rigidity of the right side of the abdomen, and the pain may also be referred to that region. It is chiefly in children that difficulty in diagnosis may arise, but the high temperature, flushed face and grunting respiration should help one to avoid mistakes. Right-sided pyelitis is a frequent source of error. The early development of a high temperature, tenderness in the renal angle and the presence of organisms in the urine should serve to differentiate it from appendicitis. Cholecystitis should not often give rise to any difficulty in diagnosis. In a case of peritonitis it may not be possible to diagnose its origin, whether from appendicitis, diverticulitis, salpingitis, or from a primary pneumococcal, streptococcal, gonococcal, or acute tuberculous infection. Regional ileitis is another uncommon source of error. On several occasions, bleeding from a ruptured Graafian follicle has led me into making a diagnosis of acute appendicitis. An appendix abscess may have to be distinguished from pyonephrosis or perinephric abscess, carcinoma of the cæcum, an inflamed retained testis, suppurating dermoid of the ovary, or from pyosalpinx.

### TREATMENT.

The vast majority of cases of appendicitis are best treated by immediate operation. Every case should be seen by a competent surgeon at the earliest opportunity, and it should be left to him to decide whether operation is called for or not. The only common case in which delay is not only permissible but advisable is one in which symptoms have been present for several days but the patient is not growing worse, and all the signs point to a strict localization of the inflammation. In such a case dense adhesions may add very greatly to the difficulties and dangers of operation. If left alone, either spontaneous resolution occurs and the case may be dealt with a month or two later, or a localized abscess forms which may be drained when it has become sufficiently accessible.

Where there is any doubt as to the diagnosis or when the mischief is situated in the pelvis, a right paramedian incision is advisable. In other cases I

consider McBurney's muscle-splitting incision the best. It is quickly made and easily closed, it enables one to approach the appendix from the outer side of the cæcum and so to avoid exposure of the small gut; if a drain is needed, it is kept well away from the ileum and so does not tend to cause intestinal adhesions. The incision should be kept as high as possible, for though it is easy to pull up a low-lying appendix, a high or retrocæcal appendix is not easily pulled down. If more room is required, it can be converted into a Rutherford-Morrison incision, or a better-placed one can be made.

Should extensive peritonitis be present, I have for many years past removed the appendix without ligating the stump, and through it have pushed a catheter into the cæcum, fixing it by means of a purse-string suture. Any pus which forms can escape alongside the catheter, while the catheter itself is used to administer saline by the continuous drip method. It may also be used to wash out the bowel or to get rid of gaseous distension. Fluid is absorbed just as well as when given intravenously, and the drip can be continued for days if necessary; any excess of fluid merely acts as an aperient. I have never known ileus, that bugbear of peritonitis, to develop in a case treated on these lines. In my opinion this device, now extensively practised, is the greatest advance of recent years in the treatment of the worst cases of appendicitis; it has saved many lives in my own practice.

### SUMMARY.

There are two points in particular which I wish to stress if the present mortality from acute appendicitis is to be lowered. The first is the necessity for earlier diagnosis before signs of peritonitis have developed.

In many instances this can only be effected by rectal examination. My second point is that when operating upon any advanced case, other than a localized abscess, in which drainage is required, a catheter should be inserted into the cæcum and saline should be administered through this. It promotes a speedy convalescence, and is undoubtedly a potent life-saving measure.

